# Case Report: A case of early rapid recurrence of a primary intracranial alveolar soft part sarcoma with high proliferative activity and complex molecular variations

**DOI:** 10.3389/fonc.2026.1943604

**Published:** 2026-10-15

**Authors:** Xinying Ma, Jing Liu, Defu Yang, WanLiang Sun, Ying Xu

**Affiliations:** 1 Department of Radiation Oncology, General Hospital of Northern Theater Command, Shenyang, China; 2 Oncology Department, No. 968 Hospital of the Joint Logistics Support Force of the People’s Liberation Army, Jinzhou, China

**Keywords:** ASPSCR1-TFE3 gene fusion, BRAF V600E mutation, early recurrence, high Ki-67 proliferation index, primary intracranial alveolar soft part sarcoma

## Abstract

Alveolar soft part sarcoma (ASPS) is a rare type of soft tissue sarcoma; ASPS originating in the central nervous system is even rarer. Previous literature has generally described it as having an indolent course with a favorable prognosis following surgical resection. This report presents a case of a 31-year-old woman with primary intracranial alveolar soft part sarcoma (PIASPS) that exhibited the opposite characteristics, demonstrating high invasiveness. The patient underwent gross total resection during the initial surgery, and postoperative histopathological examination and immunohistochemistry confirmed the diagnosis of PIASPS. Rapid local recurrence occurred just over two months after surgery; the tumor exhibited abnormally high proliferative activity, with an initial Ki-67 index of approximately 65%, which rose to 75% following recurrence. Molecular testing also revealed a complex mutation profile, including a *BRAF* p.V600E mutation, a *TP53* p.D281E missense mutation, a *TERT* promoter mutation, an *NBN* truncating mutation, an *HGF* nonsense mutation, and an *RPSAP52-HMGA2* fusion; these molecular events may have contributed to the rapid tumor recurrence, although their individual and combined effects require further functional validation. Following recurrence, the patient underwent a second surgery, followed by adjuvant radiotherapy and concurrent oral anlotinib; however, the patient died two months following the completion of radiotherapy, with an overall survival of eight months. Characterized by rapid early postoperative recurrence, sustained high proliferative activity, and complex molecular features, this case challenges the traditional perception of PIASPS as an indolent tumor. This case suggests that a high Ki-67 index combined with molecular subtyping may help identify potentially aggressive subtypes; its value in guiding decisions regarding adjuvant therapy may be further explored in future studies.

## Introduction

Alveolar soft part sarcoma (ASPS) is a malignant soft tissue tumor of unknown histological origin. It is rare overall, accounting for approximately 0.5% to 1% of soft tissue sarcomas (1). ASPS primarily affects children and young adults and typically arises in the deep soft tissues of the extremities (2). Primary intracranial alveolar soft part sarcoma (PIASPS) is extremely rare, with only case reports documented to date (3, 4). Due to its rarity, PIASPS is often misdiagnosed as more common conditions such as high-grade gliomas, meningiomas, or metastases, posing a significant clinical diagnostic challenge (5, 6). The pathological diagnosis of PIASPS primarily relies on characteristic organoid or alveolar structures and TFE3 nuclear immunopositivity, the latter serving as a surrogate marker for the *ASPSCR1-TFE3* gene fusion (7). Previous studies based on sporadic cases and small case series have shown that PIASPS typically follows a relatively indolent clinical course, and its prognosis is often considered favorable (8, 9). However, due to a severe lack of accumulated case data, current understanding may only represent a partial picture of the tumor’s biological profile. Whether aggressive subtypes exist, as well as the corresponding clinical-pathological characteristics and molecular driving mechanisms of such subtypes, remain to be systematically elucidated and urgently require in-depth investigation based on a larger and more diverse set of case data. We report a case of PIASPS that recurred rapidly after surgery, in stark contrast to its typically indolent clinical course (10). The most prominent pathological feature of this case was a significantly elevated Ki-67 proliferation index, which reached 65% at initial diagnosis and rose to 75% at recurrence; this dynamic change constitutes a quantitative indicator of the tumor’s aggressive behavior. Comprehensive molecular profiling based on next-generation sequencing identified a series of complex mutational events, including the *BRAF* p.V600E hotspot mutation, the *TP53* p.D281E missense mutation, *TERT* promoter mutations, *NBN* truncating mutation, HGF nonsense mutation, and an *RPSAP52-HMGA2* fusion gene. Although some of these mutations are common in high-grade malignant tumors, the coexistence of this combination of mutations in a single PIASPS case has not been previously reported in the literature. This case suggests that PIASPS can present as a highly aggressive variant and underscores the potential value of incorporating the proliferation activity index and molecular subtyping results into a comprehensive evaluation system to improve prognostic assessment and identify potential therapeutic targets.

## Case description

The patient is a 31-year-old woman (age at the initial visit and at the time of the two surgeries) with no significant past medical history; she reported no history of hypertension, diabetes, or coronary heart disease. The complete clinical course is shown in **Figure 1**. On August 1, 2024, she presented to a local hospital with a 3-day history of dizziness and intermittent throbbing headaches that occurred without any apparent cause. A cranial MRI revealed a space-occupying lesion in the left temporal lobe, and she was referred to our hospital for further evaluation and treatment. On admission, the patient was alert and oriented, with no abnormalities on neurological examination. A non-contrast and contrast-enhanced cranial CT scan performed on the same day revealed a nearly circular lesion of mixed density in the left frontal-parietal-temporal region, measuring approximately 4.3 cm × 4.2 cm (**Figures 2A, B**). A cranial MRI further revealed a well-defined, encapsulated mass in the left frontal-parietal-temporal region with heterogeneous T1 and T2 signal intensity (**Figures 2C, D**). Low-signal areas were visible around the periphery and in the center of the lesion, accompanied by a liquid-liquid interface, suggesting fresh hemorrhage and hemosiderin deposition. Extensive edema was present around the lesion, causing compression and flattening of adjacent sulci and fissures; the left lateral ventricle was compressed, and the midline was shifted to the right. The lesion appeared hyperintense on diffusion-weighted imaging (DWI) sequences (**Figure 2E**) and hypointense on the corresponding apparent diffusion coefficient (ADC) map (**Figure 2F**), suggesting restricted diffusion. Contrast-enhanced imaging showed partial capsular enhancement with marked heterogeneous internal enhancement (**Figure 2G**). Additional punctate T1-isointense and T2-hyperintense signals were visible in the bilateral frontal lobes and the left corona radiata, appearing as high signal on the T2 FLAIR sequence (**Figure 2H**). The preoperative radiological impression was a left frontal-parietal-temporal lesion, highly suspected to be a glioma.

**Figure 1:**
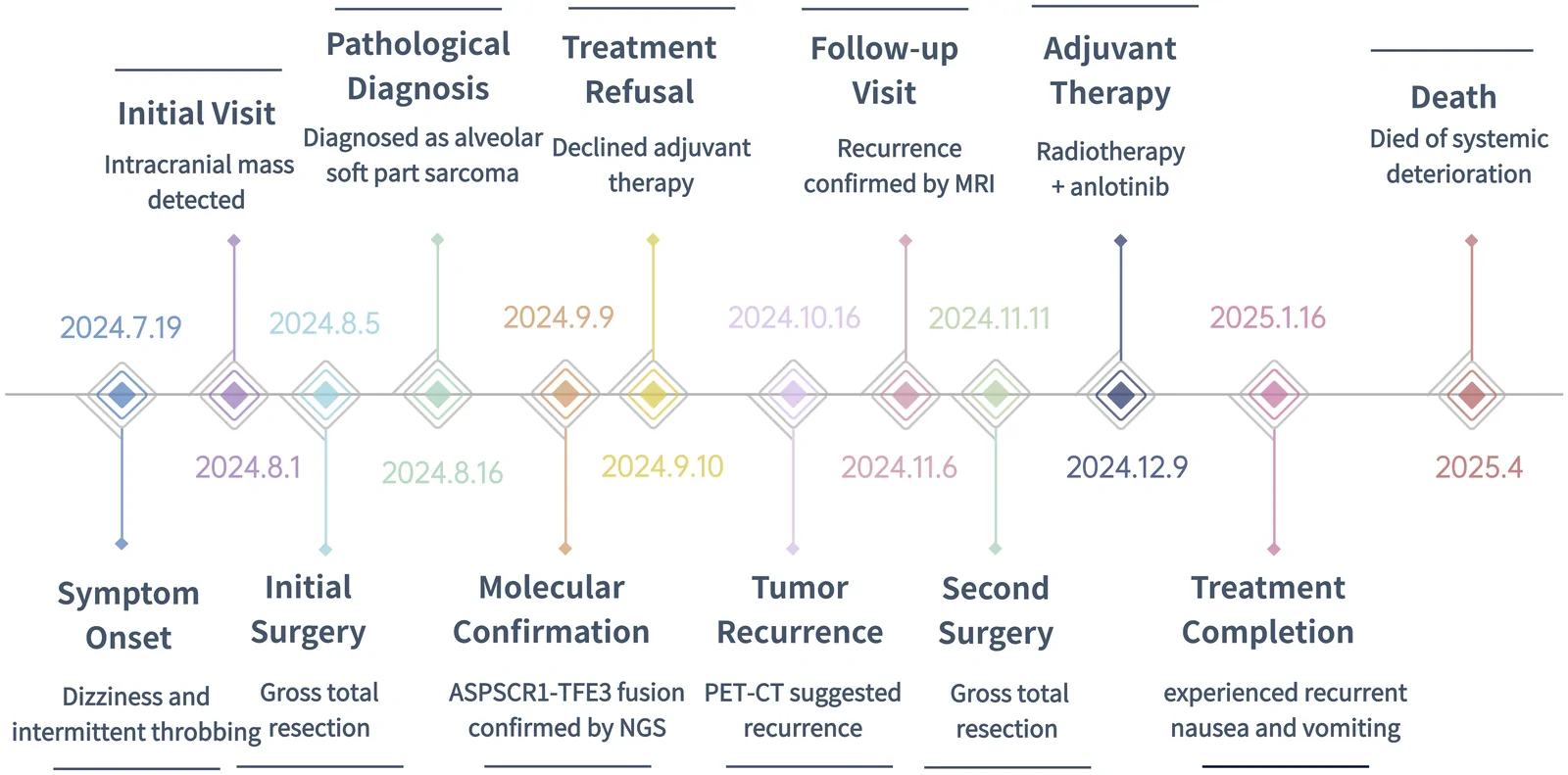
Timeline of the patient’s clinical course.

**Figure 2:**
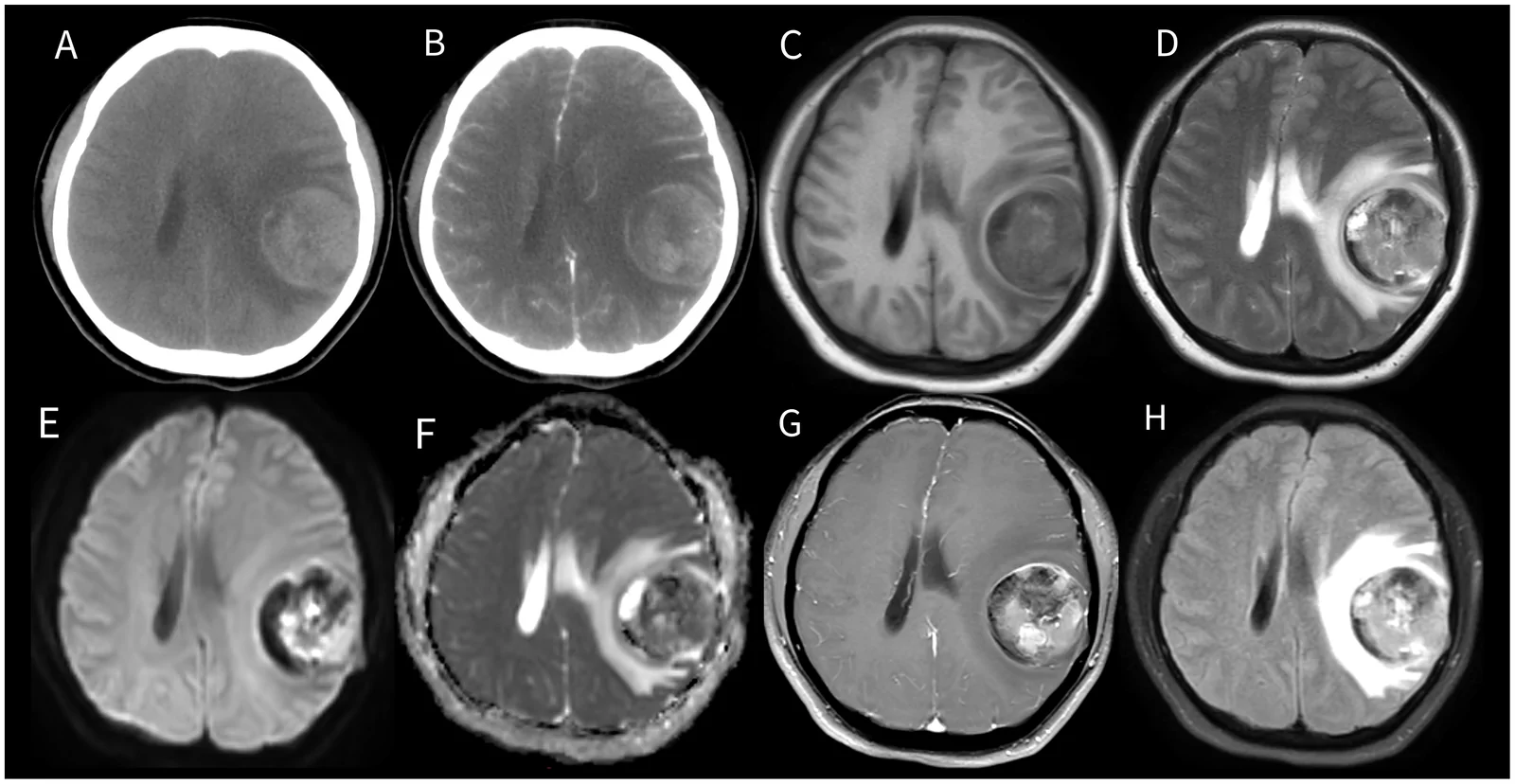
Preoperative images of a left frontotemporal lobe mass. **(A)** Non-contrast head CT; **(B)** Contrast-enhanced head CT; **(C)** T1-weighted MRI; **(D)** T2-weighted MRI; **(E)** Diffusion-weighted imaging (DWI); **(F)** Apparent diffusion coefficient (ADC) image; **(G)** Contrast-enhanced T1-weighted MRI; **(H)** T2 FLAIR sequence.

On August 5, 2024, the patient underwent craniotomy of the left frontal-parietal-temporal lobes, during which the tumor was macroscopically resected in its entirety, the dura mater was repaired, and the bone flap was repositioned. Intraoperatively, the tumor appeared grayish-red, with a consistency resembling fish meat; it was well-vascularized and was soft to firm in consistency. The surgical record indicated “complete resection” of the tumor, suggesting that gross total resection (GTR) was achieved based on the intraoperative assessment. Follow-up CT and MRI scans performed within 24 hours postoperatively showed no clear evidence of residual tumor in the surgical cavity (**Figures 3A–F**).Postoperative follow-up involved radiological assessment of intracranial lesions using the Response Assessment in Neuro-Oncology (RANO) criteria, with disease progression determined by evaluating changes in lesions on T1-weighted contrast-enhanced sequences, T2/FLAIR edema, and neurological status.

**Figure 3:**
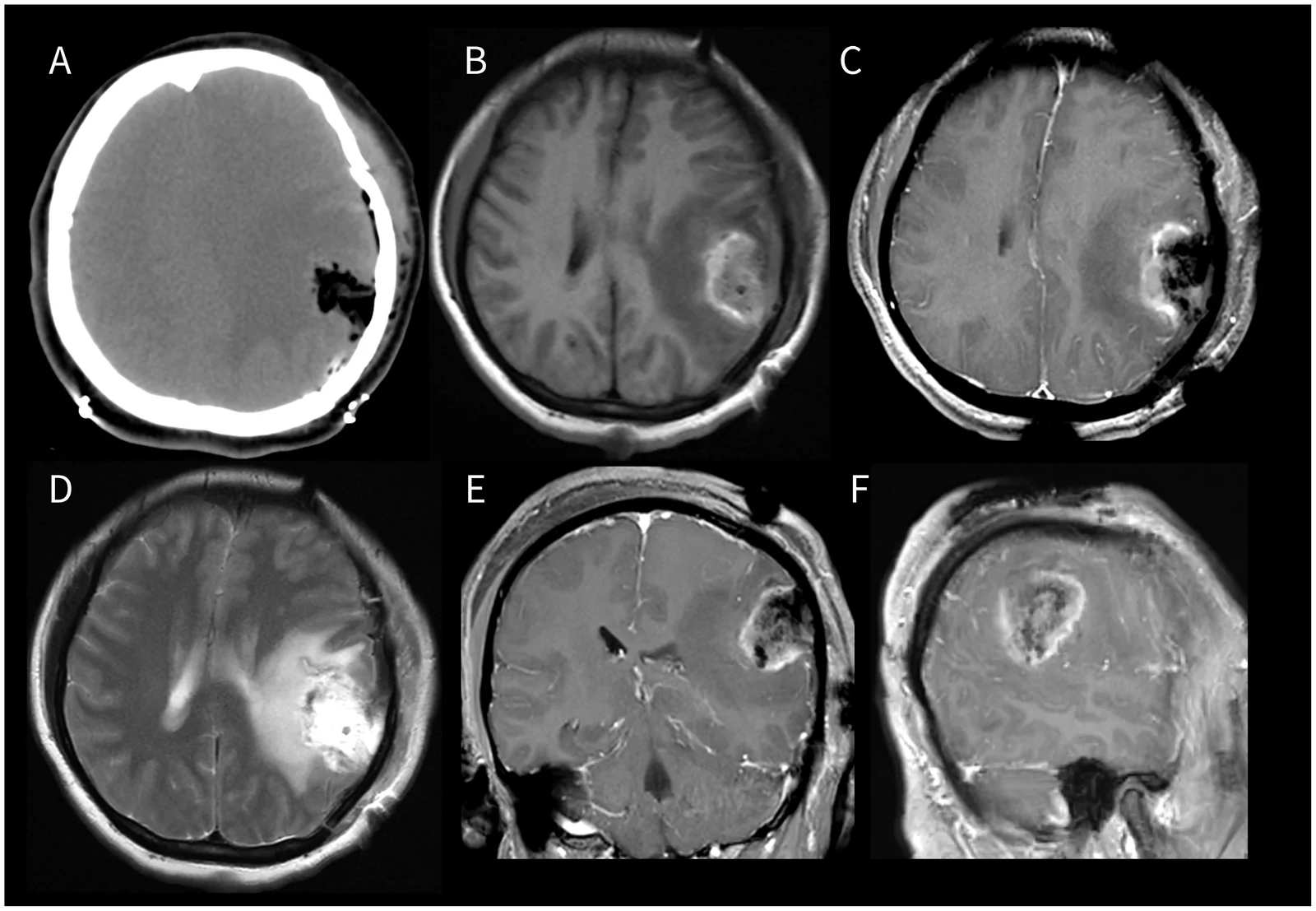
Postoperative images of a left fronto-parieto-temporal lesion resection. **(A)** Non-contrast CT scan of the head; **(B)** T1-weighted MRI; **(C)** Contrast-enhanced T1-weighted MRI; **(D)** T2-weighted MRI; **(E)** Coronal contrast-enhanced T1-weighted MRI; **(F)** Sagittal contrast-enhanced T1-weighted MRI.

Postoperative gross pathological examination revealed that the resected specimen measured approximately 4.5 × 4.5 × 1.2 cm, was grayish-yellow to dark red in color, and had a soft consistency (**Figure 4A**). Microscopically, tumor cells were arranged in nests and clusters within a highly vascularized stroma. The cells were large, with pale eosinophilic cytoplasm and round to oval nuclei exhibiting marked nuclear atypia (**Figure 5A**). Immunohistochemical staining showed that the tumor cells were positive for TFE3 (nuclear), Vimentin (Vim), INI-1, CD34, and CD68 (**Figures 5B–F**); partially positive for CD163 and S-100 (**Figures 5G, H**); focally positive for CD56 (**Figure 5I**); and only a few scattered cells were positive for Olig-2 (**Figure 5J**); GFAP, EMA, IDH1, synaptophysin, cytokeratin (CK), HMB-45, and Melan-A were all negative. The Ki-67 labeling index was approximately 65% (**Figure 5K**). Based on the characteristic alveolar architecture and strong nuclear expression of TFE3, the pathological diagnosis was a primary intracranial alveolar soft tissue sarcoma of the left frontal-parietal-temporal lobes.

**Figure 4:**
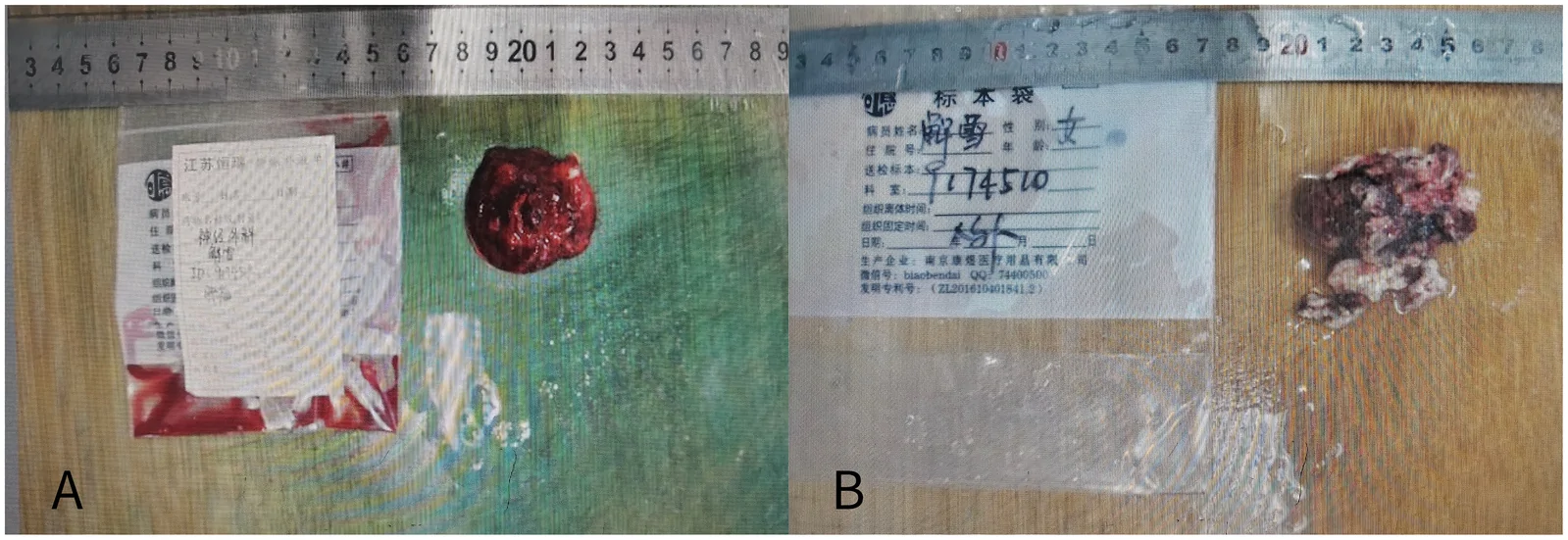
Macroscopic specimens from the first **(A)** and second **(B)** surgeries. The tumor is grayish-red, soft in consistency, and has a fish-like appearance on the cut surface **(A)**; the specimen from the second surgery is grayish-red to grayish-white, with a solid cut surface and visible areas of necrosis **(B)**. A steel ruler is placed next to the specimens.

**Figure 5:**
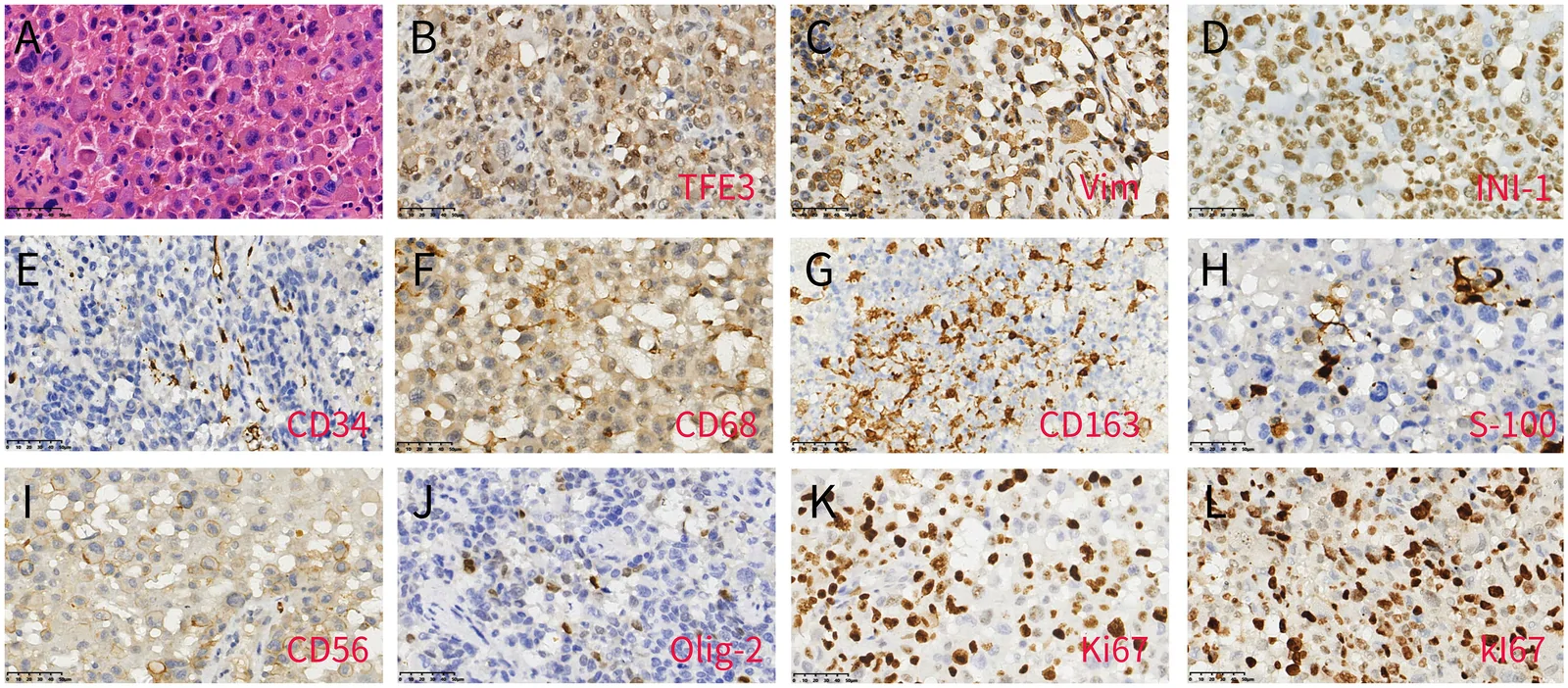
Histopathological and immunohistochemical characteristics of the tumor. **(A)** Hematoxylin-eosin staining shows tumor cells arranged in nests and clusters within a highly vascularized stroma; the tumor cells are large, with pale eosinophilic cytoplasm and round to oval nuclei exhibiting marked nuclear atypia. **(B–F)** Immunohistochemical staining showed positive expression for TFE3 (nuclear positivity), vimentin, INI-1, CD34, and CD68. **(G)** Partial positivity for CD163. **(H)** Partial positivity for S-100. **(I)** Focal positivity for CD56. **(J)** Olig-2 was positive in only a few scattered cells. **(K)** The Ki-67 proliferation index after the first surgery was approximately 65%. **(L)** Ki-67: The proliferation index after the second surgery was 75%. The original magnification for all histopathological images is ×40.

Fluorescence *in situ* hybridization (FISH) confirmed the presence of an *ASPSCR1–TFE3* gene fusion, a characteristic driver mutation of ASPS. Next-generation sequencing further identified a complex spectrum of accompanying molecular alterations: *BRAF* p.V600E (mutation frequency 44.94%), *TP53* p.D281E (49.00%), *TERT* promoter c.-124C>T (48.79%), *NBN* p.R466Kfs5 (1.11%), and *HGF* p.Q379Ter (9.85%). RNA sequencing identified an *RPSAP52* (exon 1)–*HMGA2* (exons 2–4) fusion (FFPM of 9.76). Sequencing of a germline control from peripheral blood did not detect any pathogenic or suspected pathogenic germline variants in genes such as *TP53* and *NBN*, confirming that both the *TP53* p.D281E and *NBN* p.R466Kfs5 mutations are somatic events. Germline testing also identified heterozygous variants of unknown significance (VUS), including *SDHA* p.T578N, *POLE* p.P1205L, *EXT2* c.1806 + 10C>T, and *BMPR1A* p.R20C.

Adjuvant radiation therapy, chemotherapy, or targeted therapy is routinely recommended following surgery for high-grade sarcomas; however, the patient and her family explicitly refused postoperative radiation therapy, chemotherapy, immunotherapy, and interventions targeting the *BRAF* p.V600E and PARP inhibitors. A follow-up whole-body PET-CT scan performed approximately two months postoperatively revealed a slightly hyperdense lesion with focal hypermetabolism within the surgical bed of the left frontal-parietal-temporal lobes, suggesting a possible tumor recurrence (**Figures 6A, B**). No definite hypermetabolic abnormalities were observed in other parts of the body, including multiple small cervical lymph nodes and a few old pulmonary lesions. Based on the physical examination, preoperative chest CT, and the patient’s history of no soft tissue masses in the limbs or trunk, primary extracranial ASPS is provisionally ruled out clinically. However, it must be acknowledged that definitive exclusion of occult primary extracranial ASPS requires long-term follow-up or an autopsy.

**Figure 6:**
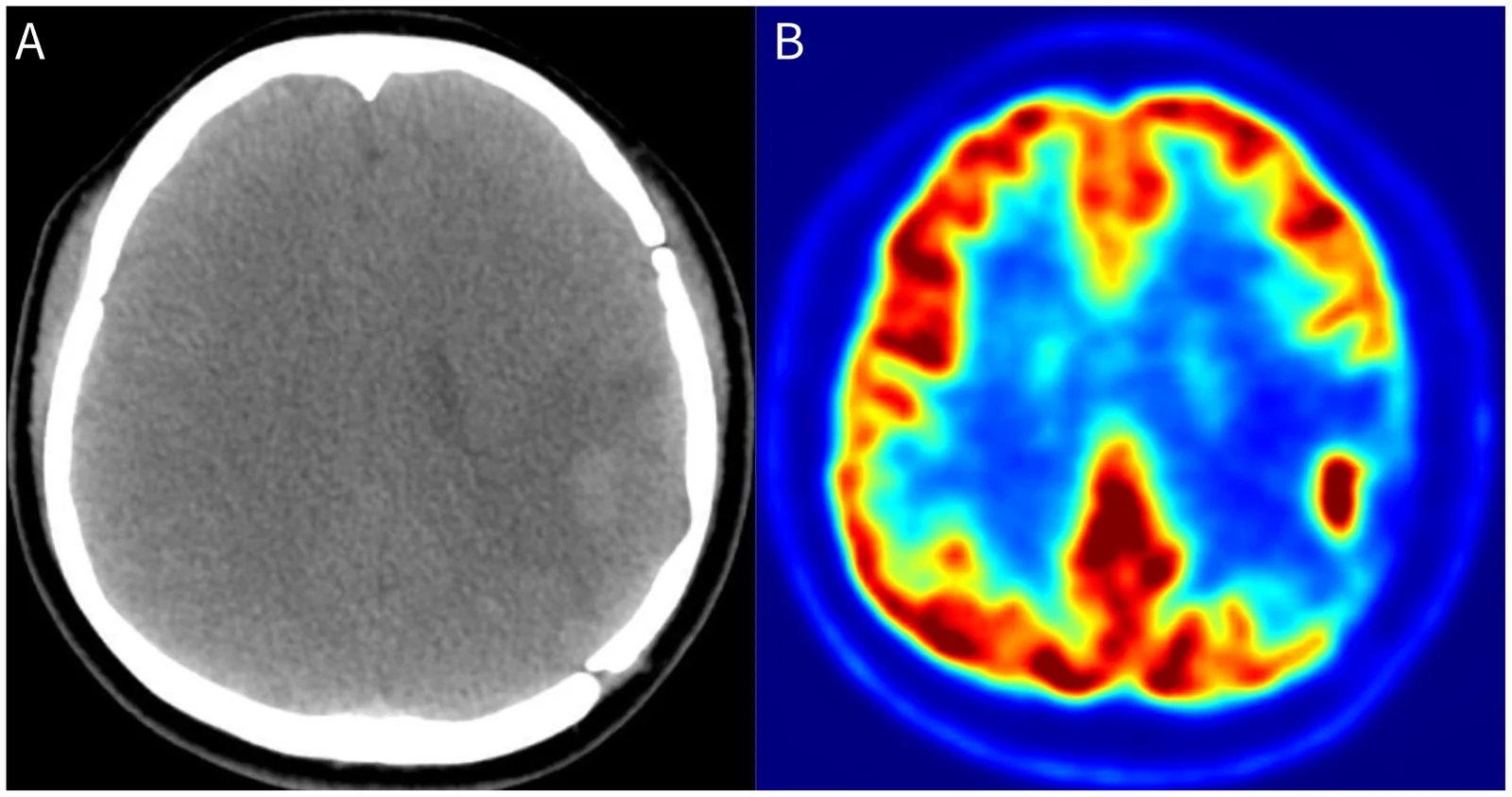
Images from the first postoperative PET-CT scan. **(A)** Unenhanced CT scan showing a slightly hyperdense lesion within the surgical bed; **(B)** The corresponding fused PET-CT image showing focal hypermetabolism in the same region.

On November 6, 2024, the patient returned for a follow-up visit due to a headache. A repeat head MRI revealed a new mass in the left temporo-parietal lobe, consistent with tumor recurrence. The images showed signal discontinuity in the postoperative bone of the left skull. Patchy abnormal signals were observed in the left temporo-parietal lobe, appearing as iso- to hypointense mixed signals on T1-weighted images, with heterogeneous enhancement and marked marginal enhancement on contrast-enhanced scans. The largest dimensions were approximately 39.4 mm × 29.8 mm, with extensive surrounding edema, compression of the left lateral ventricle, and rightward displacement of the midline structures. To manage the condition, on November 11, 2024, resection of the recurrent sarcoma in the left temporo-parietal lobe, dural repair, and craniectomy for decompression were performed under neuro-navigation and electrophysiological monitoring. Pathological examination of the second specimen revealed a tissue mass measuring 5.5 cm × 5.0 cm × 3.5 cm, gray-red to gray-white in color, and soft in consistency. Microscopically, the morphology was similar to that of the specimen from the first surgery (**Figure 4B**): tumor cells were arranged in nests, the tumor was highly vascularized, and cellular atypia was marked. Immunohistochemical findings were consistent with ASPS, with the Ki-67 proliferation index rising to approximately 75% (**Figure 5L**). Following the second surgery, the patient underwent adjuvant radiotherapy at a local hospital (total dose of 56 Gy, administered in 28 fractions of 2 Gy each), concurrently with oral administration of the multi-targeted tyrosine kinase inhibitor anlotinib. During treatment, patients experienced recurrent nausea and vomiting; no seizures, cerebral edema, or other serious adverse events were observed. After completion of radiation therapy, the patient’s general condition progressively deteriorated, manifesting as bedridden status, persistent headache, nausea, vomiting, and loss of appetite. Symptomatic and supportive treatment proved ineffective, and the patient ultimately died 2 months after the completion of radiation therapy, with an overall survival of 8 months.

## Discussion

PIASPS is clinically rare; previous case reports have generally classified it as an indolent tumor(**Table 1**). Clinically, PIASPS is typically associated with a long progression-free survival (PFS). According to a systematic review, most cases exhibit slow progression during a mean follow-up of 19.9 months (3). A meta-analysis of 24 patients reported 1-year, 2-year, and 5-year progression-free survival rates of 77.4%, 58.0%, and 41.5%, respectively (10). However, in this case, local recurrence occurred just over two months after gross total resection, which is significantly earlier than the typical progression timeline reported in the literature. Several factors may have contributed to this poor prognosis. The tumor was large, highly vascularized, and located in the left frontotemporal-parietal region, which may have led to rapid mass effect following recurrence. The patient’s refusal of adjuvant therapy after the initial surgery may also have contributed to the poor local control rate. The clinical course of rapid recurrence in this case challenges the traditional perception of this disease as indolent and further suggests the existence of highly aggressive subtypes.

**Table 1:** The 16 previous cases compared with our 1 case.

NO	Ref.	Sex/age	Location/diameter	Surgical approach	adjuvant therapy	Ki-67	Molecular detection	Salvage treatments and outcomes
1	Bodi et al. (2009)	M/39yr	L temporal/6 cm	GTR	/	/	ASPSCR1-TFE3	Tumor free; NED (10 mo)
2	Das et al. (2012)	F/17yr	Bifrontal	GTR	RT	/	/	Tumor free; NED (4 mo)
3	Mandal et al. (2013)	F/32yr	R parietal	GTR	/	/	/	/
4	Ahn et al. (2014)	F/9yr	R CPA/4.5 cm	NGTR	RT	/	/	LR (14 mo, NGTR + GKS + ICE); alive (56 mo)
5	Emmez et al. (2015) (11)	F/11yr	L frontal	GTR	RT (54 Gy)+IVA	/	t(X;17) translocation	LR (45 mo, GTR + chemotherapy); alive (54 mo)
6	Tao et al. (2016)	F/28yr	L frontal/4.1 cm	GTR	RT	/	/	Tumor free; alive (27 mo)
7	Tao et al. (2016)	M/13yr	R temporal/3.7 cm	NGTR	RT	/	/	LR (14 mo); DOD (24 mo)
8	Kumar et al. (2019) (42)	M/21yr	L post. fossa	GTR	/	/	/	Tumor free; NED (8 mo)
9	Chen et al. (2022) (8)	M/24yr	R frontal/3.5 cm	GTR	RT (52 Gy)	>10%	/	Tumor free; NED (25 mo)
10	Chen et al. (2022) (8)	F/53yr	Ant. skull base/5.0 cm	NGTR	RT (46 Gy)+AI	>10%	/	LR (10 mo, surg.); AWD (36 mo)
11	Chen et al. (2022) (8)	M/21yr	R parietooccipital/4.0 cm	GTR	/	>10%	/	Metastases (15 mo); DOD (26 mo)
12	Chen et al. (2022) (8)	F/8yr	R temporal/2.8 cm	NGTR	RT	>10%	/	LR (8 mo, surg.); DOD (50 mo)
13	Chen et al. (2022) (8)	F/16yr	L parietal/3.0 cm	GTR	RT (42 Gy)	>10%	/	LR (32 mo, surg. + ICE + RT); DOD (61 mo)
14	Rao et al. (2023) (7)	M/3yr	R frontoparietal	Decompression → NGTR at recurrence	RT	3%-4%	/	LR (6 mo, NGTR + RT); stable at 36 mo with mild hemiparesis
15	Rao et al. (2023) (7)	F/33yr	L frontal + parietal + R frontal	L frontal craniotomy +excision	/	3%-4%	/	Died at 13 mo after palliative treatment
16	Rao et al. (2023) (7)	F/13yr	R parietooccipital	excision	/	3%-4%	/	Lost to follow-up
17	Present (2024)	F/31yr	L frontotemporal lobe	GTR	RT + Anlotinib	First attempt 65% → Recurrence 75%	ASPSCR1-TFE3 + BRAF V600E + TP53 + TERT + NBN + RPSAP52-HMGA2	LR (2 mo, surg.); DOD (8 mo)

ICE, ifosfamide, carboplatin, and etoposide,

IVA, ifosfamide, vincristine, and actinomycin.

AI, doxorubicin and ifosfamide.

M, Male; F, Female; R, Right; L, Left; GTR, Gross total resection; NGTR, Non-gross total resection; RT, Radiotherapy; GKS, Gamma knife surgery; NED, No evidence of disease; DOD, Dead of disease; LR, Local recurrence.

From a pathological perspective, the aggressive course of this case is closely associated with an abnormally elevated Ki-67 proliferation index and a complex spectrum of molecular alterations. In previous reports on PIASPS, the Ki-67 index was often not emphasized or was observed only at low levels (6). Rao et al. reported three cases of PIASPS, in which the Ki-67 labeling index was low (3%–4%) (7). Chen et al. reported a case with a high Ki-67 index (>10%); the patient remained recurrence-free at 25 months postoperatively following gross total resection and without adjuvant therapy (8). The aforementioned meta-analysis of 24 patients also did not highlight an association between extremely high proliferative activity and rapid disease progression (10). However, other studies have indicated that patients with elevated Ki-67 indices are suitable candidates for adjuvant radiotherapy (11), indirectly supporting the use of a high proliferative index as a high-risk warning indicator and an indication for early adjuvant intervention. In contrast, in this case, the Ki-67 index reached 65% at initial diagnosis and rose to 75% at the time of recurrence, which is significantly higher than the range reported in previous studies, suggesting that this may be a variant with high proliferative activity. In soft tissue sarcomas, recurrent tumors typically exhibit higher proliferative activity than primary tumors, with the Ki-67 index increasing 2–3-fold in some series (12). Recurrence-associated increases in Ki-67 have also been reported in other tumors, reflecting the enrichment of aggressive subclones with high proliferative potential or biological selection driven by tumor growth and postoperative microenvironmental stress (13–15). Given the limited molecular evidence for clonal evolution in ASPS, it remains unknown whether the same mechanism applies to PIASPS.

At the molecular level, the presence of a typical ASPSCR1-TFE3 fusion confirms the diagnosis of ASPS (16). This fusion is caused by the t(X;17)(p11.2;q25) chromosomal translocation and activates downstream target genes such as MET and VEGF (17), driving tumor angiogenesis and invasive growth (4, 18); this fusion directly upregulates the expression of cyclin D1, making cell proliferation highly dependent on the CDK4/6 signaling pathway; CDK4/6 inhibitors combined with anti-angiogenic drugs exhibit a synergistic effect in ASPS xenograft models (16). In addition, molecular testing identified several accompanying mutations, including *BRAF* p.V600E, *TP53* p.D281E, *TERT* promoter c.-124C>T, *NBN* p.R466Kfs5 truncation mutation, and the *RPSAP52-HMGA2* fusion. Previous reports have primarily focused on the diagnostic value of TFE3 nuclear staining and the *ASPSCR1–TFE3* fusion itself, whereas the coexistence of the aforementioned mutations in PIASPS has rarely been documented. Although these mutations are not diagnostic markers for PIASPS, their concurrent occurrence provides important clues for understanding the aggressive phenotype of this tumor and exploring potential intervention targets.

Among accompanying mutations, the *BRAF* p.V600E mutation warrants particular attention. The BRAF gene encodes the serine/threonine kinase B-raf, a key component of the RAS-RAF-MEK-ERK (MAPK) pathway, which regulates cell proliferation and differentiation under physiological conditions (19). The *BRAF* p.V600E mutation (c.1799T>A) results in the replacement of valine at codon 600 with glutamic acid, leading to constitutive activation of the kinase and sustained amplification of ERK signaling, which drives proliferation, resistance to apoptosis, and invasiveness (20). This mutation accounts for 90% of all *BRAF* mutations and occurs at high frequencies in malignant melanoma (approximately 50%), papillary thyroid carcinoma (approximately 30%–80%), colorectal cancer (approximately 10%), and non-small cell lung cancer; it is also found in pleomorphic xanthoastrocytoma (approximately 60%) and glioma (approximately 50%) (21–23). The *BRAF* p.V600E mutation is a rare event in soft tissue sarcomas; a study of 1,964 patients with advanced soft tissue sarcomas showed a detection rate of only 0.6% (11 out of 1,964 cases), including 4 cases (0.2% of the total cohort) that were malignant peripheral nerve sheath tumors. To date, there have been no case reports of the *BRAF* p.V600E mutation in ASPS or PIASPS; this case may represent the first such report. The *BRAF* p.V600E mutation predicts sensitivity to *BRAF* and MEK dual inhibitors (24), in one patient with metastatic sarcoma, treatment with dabrafenib combined with trametinib achieved complete regression of lung metastases (25).However, the patient in this case declined the relevant targeted therapies, so it was not possible to verify any survival benefit.

In addition, a *TERT* promoter mutation and an *NBN* truncating mutation were detected in this case; the *NBN* truncating mutation results in a defect in homologous recombination repair, which theoretically confers sensitivity to PARP inhibitors (26, 27).The *TERT* promoter mutation (c.-124C>T) is one of the most common non-coding region mutations in cancer. It upregulates *TERT* gene transcription by creating a new ETS transcription factor binding motif, reactivating telomerase, and thereby driving tumor cell immortalization and malignant progression (28). Its overall incidence in soft tissue sarcomas is approximately 9.5%. In chondrosarcoma, it is significantly associated with high-grade tumors, metastasis, and an aggressive course, and is considered a marker of poor prognosis (29, 30). Its predictive value for the efficacy of immune checkpoint inhibitors (ICIs) varies by tumor type: in melanoma, *TERT* mutations are associated with better responses to ICIs (31, 32); however, evidence in glioblastoma is inconsistent, with some studies suggesting a potential benefit (32); other studies have found that TERT mutations are associated with shorter progression-free survival (p=0.053) (33). Some studies have indicated that *TERT*-mutant and wild-type glioblastomas exhibit similar circulating immune microenvironments and responses to immunotherapy (34). The root cause of this contradiction may lie in the fact that T-cell infiltration in glioblastoma is inherently limited by the blood-brain barrier, combined with the high concentration of immunosuppressive cells in the tumor microenvironment, this generally limits the efficacy of immune checkpoint inhibitors (35). Therefore, further clinical data are needed to determine the prognostic significance of *TERT* mutations in PIASPS for immunotherapy.

Evidence supporting *POLE* mutations as predictive biomarkers for immunotherapy has been growing in recent years, though it primarily comes from specific tumor types such as colorectal cancer and endometrial cancer. In a colorectal cancer cohort, patients with *POLE* “loss of proofreading” (LOP) mutations achieved an objective response rate of 88.9% and a disease control rate of 100% following immunotherapy (36); another real-world, pan-cancer study also demonstrated significantly improved survival following immunotherapy in patients with *POLE*/*POLD1* mutations (37); patients with colorectal cancer harboring pathogenic *POLE* mutations may have a tumor mutational burden (TMB) of 235 mutations/Mb or higher, which is closely associated with high response rates to immunotherapy (38). However, none of the above evidence comes from ASPS or PIASPS, and there are currently no clinical data on the predictive value of POLE mutations for immunotherapy in soft tissue sarcomas. Furthermore, the *POLE* p.P1205L mutation detected in this case is a variant of VUS, which is fundamentally different from the pathogenic *POLE* mutations in the aforementioned studies; its functional impact cannot yet be determined. In this case, the TMB was 2.13 Muts/Mb and the MSI status was MSS, neither of which supports the possibility of a high response to immunotherapy. Furthermore, due to the blood-brain barrier and the locally immunosuppressive microenvironment in central nervous system tumors, the response to immunotherapy may be limited even with elevated TMB (35). Therefore, the clinical value of this *POLE* variant is currently difficult to determine, and its predictive significance for immunotherapy in PIASPS requires further investigation.

Another noteworthy finding is the *RPSAP52–HMGA2* fusion. The significance of this fusion in ASPS remains to be elucidated, but its components have been functionally linked to sarcomagenesis (39). *HMGA2* is an oncogene, and its antisense pseudogene, *RPSAP52*, upregulates *HMGA2* expression through various mechanisms, including the formation of R-loops and interaction with IGF2BP2, and constitutes the HMGA2–IGF2BP2–RAS oncogenic signaling axis. In sarcomas, high levels of *RPSAP52* expression are significantly associated with poor patient prognosis (40). These findings suggest that the *RPSAP52-HMGA2* fusion is a molecular event associated with sarcoma aggressiveness; however, its specific significance in ASPS (particularly PIASPS) remains unclear and warrants further investigation.

The aggressive course of this case is driven by a combination of clinical, pathological, and molecular factors: the clinical context is characterized by a large tumor volume, a specific location, and the absence of adjuvant therapy; persistent high Ki-67 expression reflects high proliferative activity; and the *ASPSCR1-TFE3* fusion, combined with *BRAF V600E*, *TP53*, *TERT*, *NBN*, and *RPSAP52-HMGA2* mutations, provided the molecular basis for multi-pathway synergy. The combination of these factors may explain the extremely early recurrence and poor survival in this case, and suggests that the coexistence of high Ki-67 expression and complex molecular alterations in PIASPS may serve as a reference marker for identifying high-risk subtypes.

This case provides several insights into the diagnosis and prognostic assessment of intracranial ASPS. In this case, preoperative imaging revealed a well-defined mass with marked peritumoral edema and heterogeneous enhancement; it was difficult to distinguish this lesion from a high-grade glioma, metastatic cancer, or meningioma on imaging (41). The diagnosis was ultimately confirmed through histopathological and molecular testing: histologically, the tumor exhibited characteristic acinar/nest-like structures with a rich vascular network; immunohistochemistry showed diffuse, strong nuclear positivity for TFE3; and FISH confirmed the *ASPSCR1-TFE3* gene fusion. This diagnostic pathway suggests that for young patients with highly vascularized intracranial mass lesions, TFE3 immunohistochemistry may be included to aid in the differential diagnosis of rare sarcomas such as ASPS (42). The Ki-67 index (65%–75%) was abnormally elevated. Given that a high proliferation index in soft tissue sarcomas is typically associated with greater aggressiveness and a poorer prognosis (43), we believe that incorporating the Ki-67 index into routine pathological reports and risk stratification systems for intracranial ASPS should be considered; a significantly elevated index may indicate a high-risk subtype, which could facilitate early, proactive intervention (44).

From a therapeutic perspective, this case also offers valuable lessons. Although GTR the cornerstone of treatment (45), even when intracranial sarcomas are recorded as having undergone “complete resection,” the risk of local recurrence cannot be entirely ruled out. This is because anatomical constraints within the cranium make it difficult to achieve wide surgical margins (46), and unlike the routine practice of ink-staining margins and pathological evaluation for soft tissue sarcomas of the extremities, the margin status of intracranial tumor specimens is often difficult to determine accurately (47); consequently, “complete resection” may not represent a biologically curative outcome in subtypes with high proliferative activity. Particularly for cases with significantly elevated Ki-67 indices and complex molecular subtypes, surgery alone may not be sufficient to control disease progression (44). The fact that rapid recurrence occurred after the initial surgery in the absence of adjuvant therapy suggests that, even when radiologically complete resection is achieved, a multidisciplinary discussion should be conducted as early as possible to evaluate the indications for postoperative adjuvant therapy (48).

This case identified potential therapeutic targets, including BRAF V600E, CDK4/6 pathway abnormalities, and NBN defects; however, the patient and her family explicitly refused BRAF/MEK inhibitors and PARP inhibitors immediately after the initial surgery, and targeted therapy was not administered. The decision to use anlotinib after recurrence was primarily based on the following: in ASPS, the *ASPSCR1*-*TFE3* fusion drives VEGF overexpression, and anti-angiogenic therapy has a clear biological rationale (18); furthermore, this drug has been approved in China for the treatment of soft tissue sarcoma, is administered orally, and has manageable adverse reactions (49) Unfortunately, despite receiving radiotherapy combined with the anti-angiogenic targeted therapy anlotinib following the second surgery, the patient’s tumor progression could not be halted. This suggests that short-course monotherapy with targeted agents or local radiotherapy alone may have limited efficacy in PIASPS with high proliferative activity. For PIASPS cases that demonstrate a rich blood supply on preoperative imaging and a high postoperative Ki-67 index, even after successful gross total resection supplemented by early treatment, comprehensive molecular subtyping should be performed concurrently to identify meaningful molecular markers for molecular stratification. This provides a direct basis for the early intervention of targeted therapies such as BRAF and PARP inhibitors, thereby optimizing treatment decisions and improving therapeutic efficacy.

Based on the literature, for PIASPS with high proliferative activity and *BRAF* V600E mutations, it is theoretically feasible to consider initiating treatment with BRAF/MEK inhibitors. Dabrafenib in combination with trametinib has been approved by the FDA for the treatment of solid tumors with *BRAF* V600E mutations (50, 51). Following disease progression or intolerance, treatment should be switched to anti-angiogenic TKIs, with CDK4/6 inhibitors or PARP inhibitors serving as alternative or combination strategies. The rationale for CDK4/6 inhibitors stems from preclinical studies showing that ASPSCR1-TFE3 drives high cyclin D1 expression and that blocking CDK4/6 inhibits ASPS proliferation (16); the rationale for PARP inhibitors stems from studies showing that NBN defects lead to impaired homologous recombination repair and that HRD characteristics in soft tissue sarcomas predict sensitivity to PARP inhibitors (52, 53). However, direct evidence for these approaches is lacking in PIASPS, and this case did not verify their efficacy.

In summary, this case highlights the potential value of integrating histopathology, proliferation indices, and molecular subtyping for PIASPS risk stratification. A significantly elevated Ki-67 index and specific molecular alterations may be associated with a more aggressive clinical course (44, 54); however, the determination of specific thresholds for risk stratification requires further validation. The complex molecular profile of this case not only explains the rapid proliferation and early recurrence but also provides a molecular basis for exploring personalized targeted treatment strategies, such as combinations of BRAF/MEK inhibitors, PARP inhibitors, or CDK4/6 inhibits with anti-angiogenic therapy (16).

This study also has certain limitations. Given the patient’s acute onset and marked mass effect at admission, surgical intervention was urgent, precluding a multidisciplinary team (MDT) discussion of the tumor prior to surgery or following recurrence; had the MDT been involved earlier, it might have facilitated a systematic evaluation of adjuvant treatment strategies. The patient and family explicitly refused adjuvant therapy following the initial surgery, making it impossible to assess the potential benefits of radiation therapy and targeted therapy. The postoperative pathology report did not describe the margin status, making it impossible to confirm R0 resection pathologically; GTR could only be inferred based on the surgical records. The recurrent tumor specimen was not subjected to NGS testing; NGS data comparing the primary and recurrent tumors would have helped elucidate clonal evolution and mechanisms of acquired drug resistance. No autopsy was performed on the patient, making it difficult to completely rule out the possibility that extracranial micrometastases contributed to the course of death. As a single-case report, the conclusions drawn here require validation through larger sample sizes.

## Conclusion

We report a case of PIASPS that recurred just over two months after the initial surgery, exhibited persistent high proliferative activity (Ki-67 65%–75%) throughout the course of the disease, and harbored complex molecular alterations, including BRAF p.V600E missense mutation, TP53 p.D281E missense mutation, *TERT* promoter mutation, NBN truncating mutation, HGF nonsense mutation, and *RPSAP52–HMGA2* fusion. The patient died approximately five months following the second surgery. This case suggests that not all PIASPS tumors are indolent; even after complete radiological resection, a significantly elevated Ki-67 proliferation index may indicate more aggressive clinical behavior. Multidisciplinary intervention should be initiated as early as possible after surgery, and comprehensive molecular subtyping may also help identify high-risk patients and could have potential value in guiding individualized targeted therapy; further research is needed in the future.

## Data Availability

The original contributions presented in the study are included in the article/supplementary material, further inquiries can be directed to the corresponding author/s.
